# US Adults’ Perceptions of Dog Breed Bans, Dog Aggression and Breed-Specific Laws

**DOI:** 10.3390/ijerph191610138

**Published:** 2022-08-16

**Authors:** Lori R. Kogan, Wendy Packman, Phyllis Erdman, Jennifer Currin-McCulloch, Cori Bussolari

**Affiliations:** 1 Clinical Sciences, Colorado State University, Fort Collins, CO 80523, USA; 2 Department of Psychology, Palo Alto University, Palo Alto, CA 94304, USA; 3 College of Education, Washington State University, Pullman, WA 99163, USA; 4 School of Social Work, Colorado State University, Fort Collins, CO 80523, USA; 5 Counseling Psychology, University of San Francisco, San Francisco, CA 94117, USA

**Keywords:** breed, breed ban, Pit Bull, dog bites, dog aggression, breed-specific laws

## Abstract

Dog aggression directed towards people is a leading reason for relinquishment and a major public health hazard. In response to the threat of dog aggression and dog bites, breed-specific legislation has been introduced in numerous cities within the United States and countries throughout the world. There is limited evidence, however, to suggest that such laws are effective. This study explored, through an online, anonymous, cross-sectional survey, US residents’ views about the bite risk of common dog breeds, breed-specific legislation, and alternative options for improved public safety. A total of 586 surveys were completed by adult US residents, 48.8% female and 48.6% male. Approximately half of the respondents reported feeling that dog bites are a serious public health issue. Although 70% of respondents were opposed to a breed ban, only 56% felt that banning specific breeds creates an animal welfare issue. Females were less likely to support a ban or agree that specific breed bans improve public safety. When participants were asked to indicate their support of several alternatives to breed-specific legislation, the most frequently endorsed options included public education about animal welfare and animal behavior, and stricter leash laws. Further research pertaining to the most effective public education dissemination methods is warranted.

## 1. Introduction

A total of 45% of US households included at least one dog in 2020, a dramatic increase from the 38% of households in 2016 [1]. The popularity of dogs is unsurprising given that having a companion dog has been shown to have a positive effect on numerous aspects of guardians’ emotional and physical health [2,3,4,5,6]. Although dogs in the home offer many benefits, they can also involve zoonotic diseases [7], injuries and accidents [8,9,10], and bite risks [11,12,13]. 

Dog aggression directed towards people is the most common canine behavior problem for which guardians seek help, one of the leading reasons for relinquishment [14,15], and a major public health hazard, especially for young children [16,17,18]. More than 4.5 million people are bitten by dogs each year in the United States, with dog bites ranking as the 13th leading cause of nonfatal emergency department visits [19,20]. Many bites, however, go unreported, meaning the actual prevalence of bites is likely to be much higher than that reflected in official sources. One study by Westgarth [21] found 25% of people report having ever been bitten by a dog, with only a fraction of these bites resulting in a hospital admissions record. 

In response to the threat of dog aggression and dog bites, breed-specific legislation (otherwise referred to as breed bans) has been introduced in the United States and numerous countries around the world. This type of legislation focuses on the banning or strict control of specific breeds deemed a danger to the general public. Although numerous dog breeds have been banned or are viewed as dangerous, one of the most commonly banned breeds is the Pit Bull, which actually consists of three separate breeds: American Staffordshire Terrier, Staffordshire Bull Terrier, and American Pit Bull Terrier. Many states allow county or city breed-specific restrictions and over 700 cities have breed restrictions [22]. However, there is limited evidence to suggest that such laws are effective. In contrast, there is growing evidence to suggest that such laws are ineffective, negatively impact animal welfare, and, in fact, do little to make communities safer [23,24,25,26,27]. There are many reasons why breed specific legislation is ineffective, including the misidentification of dog breeds, an issue that has been reported among members of the general public, animal shelter workers, law enforcement officers, and human health care professionals [28,29,30,31,32,33]. The fact that most people are unable to accurately identify dog breeds significantly impacts the ability to collect accurate breed-specific bite statistics. As a result, media stories, which influence public perception of different breeds, are often inaccurate and misleading [34,35,36,37]. Even when the breed is accurately identified, because behavior is a complex interaction of contextual and environmental factors, breed provides minimal predictive information about behavior [14,38].

A study exploring US veterinarians’ views on breed bans found only a minority feel a ban improves public safety (11%) and most (75%) feel a ban creates an animal welfare issue. Instead, veterinarians’ support alternative community policies including public education about animal behavior and welfare, stricter leash laws, and harsher penalties for dog owners in the event of a dog bite or attack [39]. Yet, despite the prevalence of breed-specific legislation in the United States and throughout the world, and the media’s depiction of ‘dangerous breeds’, little is known about how dog guardians and non-dog guardians in the United States view bite risk for common dog breeds, breed-specific legislation, and alternative options for improved public safety. Therefore, the objective of this quantitative study was to gain insights into how adults in the US view these factors: common dog breeds in terms of aggression, breed-specific legislation, and alternative public safety laws and programs. We hypothesize that participants will rate Pit Bulls as the most aggressive breed. We also hypothesize that most participants will be opposed to breed ban legislation. 

## 2. Materials and Methods

### 2.1. Sample

Survey respondents were recruited on 8 February 2022 through Prolific, an open online marketplace providing access to potential survey respondents in which survey respondents receive small monetary compensation for completing surveys (participants received $6.50 for completing this survey) [40]. The diversity of participants recruited through platforms such as Prolific is higher than that of typical Internet samples or North American college-based samples, and the quality of data collected meets standards considered acceptable in published research in the social sciences [41]. In order to minimize the influence of geographic and cultural differences on respondent data, the survey was made available only to adult (18 years or older) responders residing in the United States. The advertisement for the survey was simple, stating only that we were looking to obtain people’s opinions about certain dog breeds and laws about dog ownership.

### 2.2. Instrument

An online, anonymous, cross-sectional survey was developed using Qualtrics (Qualtrics, Inc., Provo, UT, USA). The survey was modeled after the survey used to assess veterinarians’ views of dog breeds’ bite risk and breed restrictive legislation [39]. The original survey was developed and subsequently piloted by ten veterinarians. The current survey was also piloted by six researchers external to the research team, testing for ambiguous questions, question flow, and appropriate branching. Feedback from the pilot test was assessed and, when appropriate, incorporated into the final version of the survey. The study was approved by the Colorado State University Institutional Review Board (IRB #2293). 

The survey began with an introduction that explained the purpose of the study and details designed to ensure participants had the information they needed to make informed consent to continue. These details included the fact that the survey was estimated to take approximately ten minutes to complete and there were no known risks involved in completing the survey. Participants were also given the contact information for Colorado State University Institutional Review Board and that of the primary investor. At the end of this information, participants were asked to indicate if they consented to continuing with the survey. 

The survey began with a series of demographic questions (age, gender, children), their pet status, and local breed bans. The next section asked participants to indicate their level of agreement (i.e., strongly disagree, disagree, neither agree/disagree, agree, strongly agree), with statements pertaining to general dog ownership (e.g., “An adult should be able to own any breed of dog”); and breed bans (e.g., “Banning specific breeds improves public safety”). Next, participants were asked to rate a list of common dog breeds in terms of serious bite risk from the options of ‘minimal’, ‘moderate’, ‘high risk’, or ‘don’t know’. The list was derived from the most common breeds within the United States. The last section asked about participants’ endorsement of several dog-related laws that might affect public safety (e.g., “Stricter leash laws”, “Mandatory muzzling of specific breeds when in public”). The survey was between 13 and 15 pages (separate screens), depending on branching options, and most pages had between 3–6 questions. Participants could not go back within the survey to change their answers. The survey was developed with Qualtrics tools to help prevent multiple entries from the same person and bot responses.

Statistical analyses, including descriptive statistics and chi-square, and binary logistic regression, were conducted with IBM SPSS Version 26 (IBM, Armonk, NY, USA). To analyze differences between participants based on whether they agreed or did not agree with several dog-related statements, the agreement statements were recoded into binary statements whereby ‘strongly agree’ and ‘agree’ were combined into ‘agree’, and ‘neutral’, ‘disagree’, and ‘strongly disagree’ were recoded into ‘disagree’ for analysis. Due to the number of analyses conducted, the significance level (α) was set at a more conservative level of *p* = 0.01. 

## 3. Results

A total of 586 surveys were completed by adults residing in the United States. Of the potential participants who accessed the survey, two people chose not to participate. All participants who indicated they consented to the survey completed it in its entirety. The respondents were predominantly under the age of 40 (73%), non-Hispanic/Latinx (89%), White (78%), and included 286 (48.8%) females and 285 (48.6%) males, 10 (1.7%) nonbinary, and five (0.9%) who chose to not answer. The number of respondents who reported having children was 140 (24%). There was no difference in the number of males versus females in the likelihood of having children, pet dogs, or a dog of the Pit Bull type. Approximately half (54%) owned at least one dog (Table 1). Of those who owned a dog (*n* = 319), 36 (11%) indicated the dog(s) were of a Pit Bull type. For those who indicated they did not currently have a dog (*n* = 267), 232 (87%) indicated they would consider a dog as a pet and of these (*n* = 232), 126 (54%) reported they would consider a Pit Bull type dog as a pet. All participants were asked if they had ever been bitten by a dog, to which 249 (43%) said yes. Of those bitten, 55 (22%) indicated the bite required medical attention. There was no difference in the number of males versus females in the likelihood of having children, pet dogs, a dog of the Pit Bull type, having been bitten, or having a bite that required medical attention.

### 3.1. Dog Aggression

**Figure 1:**
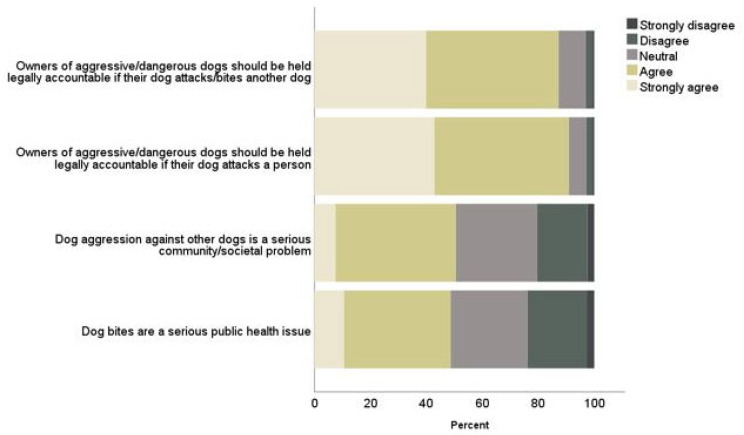
Perceptions of dog aggression.

Participants provided responses to four questions about dog aggression. Approximately half of respondents reported feeling that dog aggression against other dogs is a serious community/societal problem (51%), and that dog bites are a serious public health issue (49%). Most agreed, however, that owners of aggressive/dangerous dogs should be held legally accountable if their dog attacks/bites another dog (87%) or a person (91%) (Figure 1). No differences were found in responses based on gender, age, or child status. Of those who owned dogs, 8% (95% CI 3.5% to 13.1%) fewer, compared to non-owners, thought that owners of aggressive/dangerous dogs should be held legally accountable if their dog attacks a person (dog guardians: 278, 87%, compared to non-dog owners: 255, 96%; X^2^ = 12.34 (1), *p* < 0.001). Similarly, 7% fewer (95% CI 1.3% to 12.5%) dog guardians thought that owners of aggressive/dangerous dogs should be held legally accountable if their dog attacks another dog [(268 (84%) compared to 243 (91%) of non-dog guardians (X^2^ = 6.38 (1), *p* = 0.012)].

### 3.2. Breed and Breed Banning 

**Figure 2:**
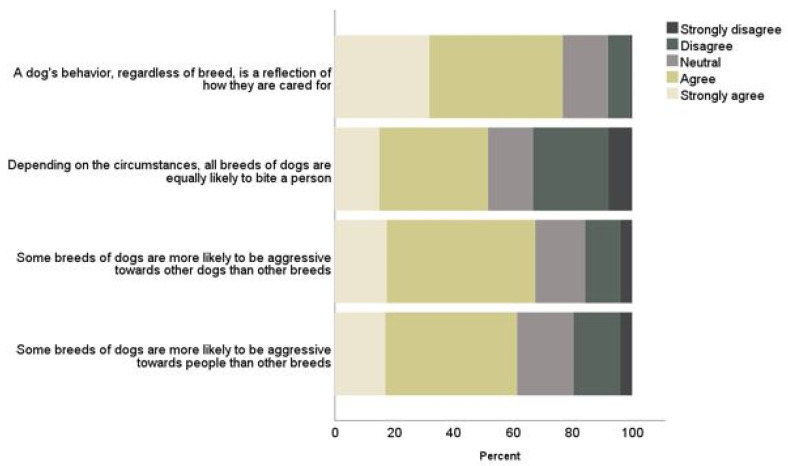
Perceived association between dog breed and behavior.

The next set of questions pertained to breed characteristics. The majority of respondents reported feeling that a dog’s behavior, regardless of breed, is a reflection of how they are cared for (77%). Most also reported feeling that some breeds of dogs are more likely to be aggressive towards other dogs (67%) or people (61%) than other breeds. Approximately half indicted they feel that, depending on the circumstances, all breeds of dogs are equally likely to bite a person (52%) (Figure 2). Twenty-one percent (95% CI 1.3% to 29.2%) more females agreed with this statement (177, 62%), compared to males (116, 41%; X^2^ = 25.65 (1), *p* < 0.001). No other differences in responses to the breed characteristics questions were found based on gender, child, or dog ownership status. 

Participants were next asked their views about breed specific legislation. Overall, participants were opposed to breed bans (398, 71%), whereas 88 (16%) supported a ban and 78 (14%) had no opinion. A binary linear regression analysis was conducted to determine predictors of support for a breed ban (yes/no). All variables were entered simultaneously. The binary regression model (Table 2) predicting support for breed ban using gender (male/female), age (under 30, 30–39 40–49,50–59, 60 and older), dog ownership, children status (yes/no), and dog bite history (yes/no) was significant (X^2^_(8)_ = 29.48, *p* < 0.001). Gender was the only significant predictor of support for a breed ban (females were less likely to support a ban; B = 0.69; *p* = 0.008).

Half of participants agreed that banning specific breeds creates an animal welfare issue (56%), and 64% felt that banning certain breeds of dogs is an overreach of governmental authority. Most also disagreed that particular dog breeds should not be allowed near children (71%) or that banning specific breeds of dogs improves public safety (82%). Seven percent (95% CI 0.1% to 13.1%) fewer females (32, 12%) reported supporting a ban than males (52, 19%); (X^2^ = 20.64 (2), *p* < 0.001); 15% (95% CI 7.1% to 22.3%) fewer females agreed that some breeds should not be allowed near children (63, 22%) compared to males (105, 37%), (X^2^ = 15.09 (1), *p* < 0.001), and 14% (95% CI 7.3% to 20.1%) fewer females agreed that specific breed bans improve public safety (30, 11%), compared to males (69, 24%; X^2^ = 18.75 (1), *p* < 0.001). Twenty percent (95% CI 11.7% to 27.5%) more females reported feeling that a ban is an overreach of governmental authority (213, 75%), compared to males (156, 55%), (X^2^ = 24.33 (1), *p* < 0.001) and 13% (95% CI 4.7% to 21.3%) more females reported feeling that banning creates an animal welfare issue (179, 63%; males: 141, 50%; X^2^ = 9.97 (1), *p* < 0.002). No differences were found based on child or dog ownership status. 

Participants were also asked two questions about dogs and dog breeds as they relate to where they choose to live. When non-dog owners (*n* = 267) were asked how a neighborhood/complex that does not allow dogs would impact their decision to live there, 139 (52%) reported it would have no impact, 85 (32%) said they would be less likely to live there and 43 (16%) said they would be more likely to live there. Additionally, all participants were asked how a potential future neighbor who owns a dog breed with an aggressive reputation would impact their decision to live in that neighborhood or complex. To this question, 388 (66%) said it would have no impact, 191 (33%) said they would be less likely to live there, and 7 (1%) said more likely to live there.

### 3.3. Pet Ownership 

**Figure 3:**
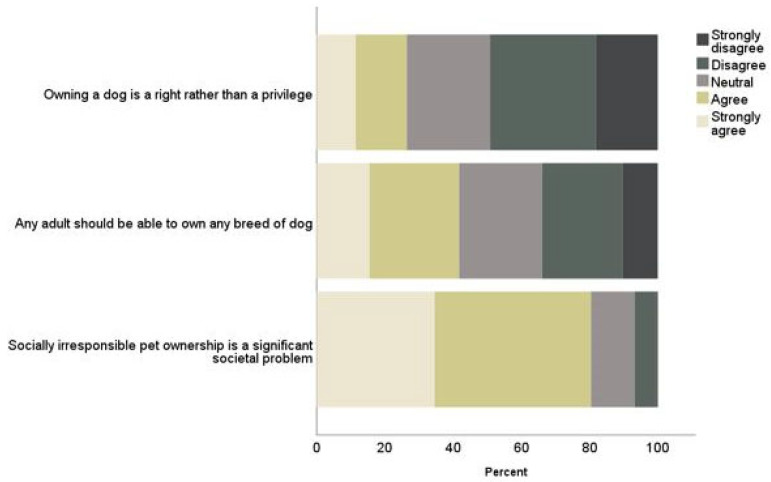
Perceptions of pet ownership.

When asked about dog ownership, the vast majority of participants (80%) agreed that socially irresponsible pet ownership is a significant societal problem. Only one-quarter of participants reported feeling that owning a dog is a right rather than a privilege, and 42% indicated they felt that any adult should be able to own any breed of dog (Figure 3). No differences based on gender were found for any of the statements. Thirteen percent (95% CI 4.2% to 22.5%) more respondents with children agreed that owning a dog is a right rather than a privilege (51, 36%) compared to those with no children (104, 23%; X^2^ = 9.41 (1), *p* < 0.002). No other differences were found based on child or dog ownership status. 

Participants were presented with a list of common breeds within the United States and asked to indicate their perception of the different breeds’ serious bite risk (defined as requiring medical treatment) as ‘minimal’, ‘moderate’, ‘high’, or ‘don’t know’. The only breed perceived by 50% or more of respondents as a high serious bite risk was the Pit Bull type (Table 3). Additional breeds reported as high risk by at least 25% of respondents included Rottweiler (43%), German Shepherd (36%), and Chihuahua (26%). The dog breeds with the lowest perceived risk of serious bites (5% or less of respondents rated them as a high risk) included Labrador Retriever (5%), Golden Retriever (5%), Yorkshire Terrier (5%), Dachshund (5%), Cocker Spaniel (4%), and Beagle (3%).

### 3.4. Community Policies 

Participants were asked to indicate their support of several policies communities have enacted in an effort to increase public safety. The most commonly endorsed policies include public education about animal welfare (67%), animal behavior (66%), and stricter leash laws (58%) (Table 4).

## 4. Discussion

Due to the frequency of dog bites in the United States, this study was designed to investigate United States’ adults’ perceptions of dog aggression, breed-specific legislation, and alternative community interventions in an effort to better understand the public’s perceptions of these key factors related to dog bites. The 586 participants in this study were predominately White, under 40 years of age, with no children. Approximately half of respondents reported owing a dog, 11% of which owned a Pit Bull. Most people who did not own a dog indicated they would consider a dog, and over half of these individuals would consider owning a Pit Bull. When queried about dog aggression and dog bites, only half of the participants reported feeling these issues are a serious community/societal problem or public health issue. Given the prevalence of dog bites, especially for young children, this suggests the need for public education about dog aggression. When asked to rate common breeds on serious bite risk, Pit Bulls were, by far, the breed rated highest, followed by Rottweilers, German Shepherds, Chihuahuas, and Doberman Pinschers. Yet Chow Chows were rated as high by only 18%, Huskies by 16%, Akitas by 12%, and Belgian Malinois by 13%. This can be contrasted with perceptions of small animal veterinarians who rated Chow Chows, Chihuahuas, German Shepherds, Rottweilers, Akitas, and Belgian Malinois as high risk (rated as high by 40% or more of participants) [39]. Other breeds rated as minimal risk by participants (e.g., Dalmatians, Cocker Spaniels) were viewed as higher risk by veterinarians [39]. 

Furthermore, only 52% of respondents reported feeling that, depending on the circumstances, all breeds of dogs are equally likely to bite a person, and only 24% felt that a dog’s behavior, regardless of breed, is a reflection of how they are cared for. Yet, due to numerous factors, there are little data to support the premise that breed is a good predictor of aggression. As Webster and Farnworth [33] note, the environment and a dog’s personality traits are better predictors of aggressive behavior than breed. This could help explain why dogs on banned lists do not cause the largest proportion of bites [42,43]. Additionally, bite proclivity by breed is difficult to calculate because information pertaining to breed populations within specific areas is typically unknown [44]. 

Instead of breed, other factors that have been shown to contribute to the likelihood of bite risk include dog attributes (e.g., male, unneutered), and negative dog/human interactions, such as chaining a dog in the yard, inadequate socialization, or harassing/teasing a dog [28,38,43,44,45,46,47,48,49]. Regardless of breed, the vast majority felt that owners of aggressive dogs should be held legally accountable if their dog attacks/bites another dog or person, although dog guardians were less inclined to support this premise. Yet, despite the number of stories in the media of stray aggressive dogs, most dog bites occur in or near the home by a dog known to the child and/or family [20,50].

Although the majority (71%) of participants opposed breed bans, males were less likely to oppose breed bans than females. Of all participants, however, only 56% felt bans create an animal welfare issue and nearly one in five reported feeling that that banning of specific breeds of dogs improves public safety. This can be compared to veterinarians’ views, in which 85% opposed breed bans and 75% felt they create an animal welfare issue [39]. In our study, females were less likely than males to support a breed ban, or to think bans improve public safety or that some breeds should not be allowed near children. 

Despite opposition by many to breed-restrictive legislation, bans are still present in many cities and counties throughout the United States, with Pit Bulls often the banned breed [51]. This is problematic for several reasons. One central issue is that the Pit Bull is not actually a breed, but instead a group of breeds. The term ‘pit bull’ typically includes American and English Bulldogs, Staffordshire Bull Terriers, American Staffordshire Terriers and American Pit Bull Terriers, in addition to mixes of these and other breeds [31]. The determination of ‘pit bull’ is typically based on a dog’s physical resemblance to one of several breeds that have been associated with the term ‘pit bull’ [32,52]. Yet, most people, even animal professionals (e.g., breeders and animal shelter staff) are unable to accurately identify a Pit Bull [30,32,33,53,54,55]. Even human health care professionals’ reports about dog aggression are often inaccurate [28], with Pit Bulls often mis-identified and inaccurately labeled as dangerous [52,56]. A study by Bykowski and colleagues found that the breed of the involved dog is missing or assumed based on phenotypic characteristics in over 50% of dog bite medical reports for children, leading to compromised validity [57]. Another challenge is that more than half of the dogs in the United States are of mixed breed [58], yet most dog bite reports include only one breed [52]. These errors carry serious implications. For example, this is especially problematic when media stories about dog bites place an emphasis on dog breed [59]. Patronek [52] found that the breed involved in dog bite incidents reported in the media frequently differs when compared to animal control reports. Yet, even the CDC’s report on Dog Bite-Related Fatalities From 1979–1988 relied on media stories in which breed identification was based on physical appearance and stereotypes of Pit Bulls [60,61,62,63]. 

Likely due to numerous factors, several studies that have assessed the impact of dog breed bans have found them ineffective [43,64,65,66,67,68,69]. Additionally, breed bans have been described as expensive and difficult to implement, and to negatively affect canine welfare since many seized dogs spend long periods of time in kennels even if they have not specifically been involved in an incident [27,42,70,71,72].

When asked about alternative community policies instead of breed bans, the interventions rated highest included public education about animal welfare and animal behavior, and stricter leash laws. Approximately half of respondents also endorsed harsher penalties for dog owners in the event of a dog bite or attack, stricter laws about picking up dog waste, and stricter fencing or containment laws. These commonly endorsed community policies mirror those supported by veterinarians, although veterinarians reported even higher levels of endorsement [39]. 

The support for public education is perhaps unsurprising given that 80% of participants reported feeling that socially irresponsible pet ownership is a significant societal problem and over 50% disagreed with the sentiment that any adult should be able to own any breed of dog. The call for educating the public about dogs in general, and dog breeds and bite risk prevention in particular, has been voiced by both animal and human medical professionals alike [73,74,75,76,77]. It would appear the need is there; one recent study found that 70% of United States children have never received dog bite education and 88% of parents wish their children were educated about bite risks [78].

There are numerous dog bite prevention programs that teach children how to interact with unfamiliar dogs [13,79,80,81] or recognize potential risk factors with a family dog [82,83]; fewer programs address how to recognize and interpret specific dog body language including dogs’ behavioral responses and their stress signals [74]. Yet, an understanding of species-specific signaling and stress signs are critical in supporting positive human/dog interactions, especially in a home with young children [83,84,85]. 

Education about animal behavior can take many forms and, although many educational efforts target children, some studies have suggested that targeting caregivers rather than young children may be of equal or greater value [78,86,87]. One type of education that has been found helpful in promoting safer behaviors around dogs and positive human–animal interactions is hazard perception training [74,88], which may include some aspect of the ladder of aggression theory suggesting that dogs typically exhibit stress-related behaviors before snapping, growling, or biting [89]. 

Closely linked to education about animal behavior is animal welfare. Educational programs about animal welfare can foster positive relationships between dogs and people, reduce bite risk, and improve canine welfare [80,90,91]. More research exploring the impact of educational programs, for both children and adults in the areas of bite prevention and welfare, are needed to determine the best mode of delivery and presentation of the material to make a positive impact on subsequent dog/human interactions. 

Limitations to the current study are those inherent in online surveys and these results cannot be generalized to the general United States population. Additionally, the survey questions pertained to participants’ self-reported opinions and perceptions, both of which have the potential to be biased. The topic of the survey may have impacted those who chose to participate, further leading to the potential for a biased sample. Further research pertaining to the public’s views of breed ban legislation and dog aggression is needed to better understand this challenging issue. 

## 5. Conclusions

Dog bites and dog aggression are serious public health concerns and create a significant burden on emergency surgical resources [50,92]. In the United States, approximately 4.5 million dog bites occur annually and 20% of these bites become infected [93]. Dog bites rank as the 13th leading cause of nonfatal emergency department visits in the United States [20] and over 1000 people in the United States are treated in hospital emergency departments for nonfatal dog bite-related injuries daily [94]. Children are at particular risk; dog bites are one of the most common causes of non-fatal injury among children [95]. Unfortunately, the number of pediatric emergency visits due to dog bites has increased recently, felt to be associated with COVID-19 health restrictions resulting in children’s increased time at home [96]. Yet, since many bites go unreported, even these disturbingly high numbers are likely an underestimate of the actual prevalence [21].

In summary, this study found that most participants do not support a breed ban, but instead endorse education about animal welfare and behavior. This is in spite of the fact that one-third of respondents said that they would be less likely to live in a home with a neighbor who owns a dog breed with an aggressive reputation. This contradiction appears to reflect the complexity of this topic and suggests that many people struggle with conflicting feelings about dog breeds and their ability to predict aggression. Yet, the majority of participants in this study appear to understand that breed is a poor predictor of aggressive behavior and are open to alternative interventions that place more responsibility on dog guardians. These sentiments bode well for both canine welfare and public safety.

## Figures and Tables

**Table 1:** Participant demographics.

Category		N (%)
Pets		
	Dog	319 (54)
	Cat	233 (40)
	Bird	21 (4)
	Reptile	26 (4)
	Small mammal	31 (5)
	Fish	21 (4)
	Other	9 (2)
Gender		
	Female	286 (49)
	Male	285 (49)
	Non-binary	10 (2)
Race		
	American Indian or Alaska Native	3 (1)
	Asian	50 (9)
	Black or African American	37 (6)
	Native Hawaiian or Pacific Islander	2 (<1)
	White	461 (80)
Ethnicity		
	Hispanic/Latinx	65 (11)
	Non-Hispanic/Latinx	521 (89)
Age		
	Under 30	261 (45)
	30–39	166 (28)
	40–49	81 (14)
	50–59	44 (8)
	60 and older	34 (6)
Children		
	Yes	140 (24)
	No	446 (76)

**Table 2:** Results of the binary logistic regression model predicting support of breed ban as a function of child status, gender, dog ownership, bite status, and age.

Variable	Coefficient (B)	Std. Error	Wald	Sig.	95% CI Lower	95% CI Higher
Children	−0.68	0.29	5.50	0.019	0.29	0.89
Gender	0.69	0.260	7.13	0.008	1.20	3.32
Dog ownership	0.51	0.25	4.04	0.044	1.01	2.74
Bite status	−0.44	0.270	2.71	0.100	0.38	1.09
Age			12.14	0.016		
Under 30	0.35	0.34	1.09	0.296	0.74	2.74
30–39	−0.09	0.39	0.05	0.822	0.43	1.95
40–49	−0.41	0.45	0.83	0.361	0.28	1.60
50–59	−1.27	0.45	7.88	0.005	0.12	0.68
60 and older	−0.68	0.29	5.50	0.019	0.29	0.89

**Table 3:** Serious Bite Risk Perception of Common Dog Breeds in the United States.

Breed	Minimal *n* (%)	Moderate *n* (%)	High *n* (%)	Don’t Know *n* (%)
Pit Bull type	75 (12.8)	194 (33.1)	293 (50.0)	24 (4.1)
Rottweiler	93 (15.9)	207 (35.3)	250 (42.7)	36 (6.1)
German Shepherd	119 (20.3)	230 (39.2)	212 (36.2)	25 (4.3)
Chihuahua	230 (39.2)	183 (31.2)	151 (25.8)	22 (3.8)
Doberman Pinscher	146 (24.9)	223 (38.1)	139 (23.7)	78 (13.3)
Mastiff	208 (35.5)	166 (28.3)	106 (18.1)	106 (18.1)
Chow Chow	219 (37.4)	145 (24.7)	104 (17.7)	118 (20.1)
American Bulldog	224 (38.2)	207 (35.3)	95 (16.2)	60 (10.2)
Siberian Husky	221 (37.7)	223 (38.1)	91 (15.5)	51 (8.7)
Boxer	209 (35.7)	241 (41.1)	74 (12.6)	62 (10.6)
Belgian Malinois	166 (28.3)	116 (19.8)	74 (12.6)	230 (39.2)
Akita	179 (30.5)	166 (28.3)	71 (12.1)	170 (29.0)
English Bulldog	276 (47.1)	178 (30.4)	68 (11.6)	64 (10.9)
Dalmatian	272 (46.4)	205 (35.0)	58 (9.9)	51 (8.7)
Great Dane	288 (49.1)	189 (32.3)	57 (9.7)	52 (8.9)
Jack Russell Terrier	309 (52.7)	164 (28.0)	43 (7.3)	70 (11.9)
Standard Poodle	356 (60.8)	153 (26.1)	37 (6.3)	40 (6.8)
Labrador Retriever	368 (62.8)	145 (24.7)	28 (4.8)	45 (7.7)
Golden Retriever	396 (67.6)	143 (24.4)	27 (4.6)	20 (3.4)
Yorkshire Terrier	364 (62.1)	130 (22.2)	27 (4.6)	65 (11.1)
Dachshund	383 (65.4)	106 (18.1)	27 (4.6)	70 (11.9)
Cocker Spaniel	386 (65.9)	107 (18.3)	21 (3.6)	72 (12.3)
Beagle	412 (70.3)	118 (20.1)	14 (2.4)	42 (7.2)

**Table 4:** Participants’ endorsement of community policies enacted in efforts to increase public safety.

Policy	Endorsement Rate *n* (%)
Public education about animal welfare	394 (67)
Public education about animal behavior	384 (66)
Stricter leash laws	339 (58)
Harsher penalties for dog owners in the event of a dog bite or attack	337 (58)
Stricter laws about picking up dog waste	331 (57)
Stricter fencing or containment laws	278 (47)
Anti-chaining and anti-tethering laws	251 (43)
Compulsory owner dog training	249 (43)
Mandatory registration for specific breeds	163 (28)
Mandatory spaying/neutering for specific breeds	147 (25)
Mandatory muzzling of specific breeds when in public	98 (17)

## Data Availability

Data available by request.
